# Belonging, believing, bonding, and behaving: the relationship between religion and business ownership at the country level

**DOI:** 10.1007/s00191-016-0447-7

**Published:** 2016-02-19

**Authors:** Brigitte Hoogendoorn, Cornelius A. Rietveld, André van Stel

**Affiliations:** 1 Erasmus School of Economics, Erasmus University Rotterdam, P.O. Box 1738, 3000 DR Rotterdam, The Netherlands; 2 Trinity Business School, Trinity College Dublin, Dublin, Ireland; 3 Kozminski University, Warsaw, Poland

**Keywords:** Compendia, Entrepreneurship, Religion, Social capital, Values

## Abstract

This cross-country study adopts a competing theories approach in which both a value perspective and a social capital perspective are used to understand the relation between religion and a country’s business ownership rate. We distinguish among four dimensions of religion: belonging to a religious denomination, believing certain religious propositions, bonding to religious practices, and behaving in a religious manner. An empirical analysis of data from 30 OECD countries with multiple data points per country covering the period 1984–2010 suggests a positive relationship between religion and business ownership based on those dimensions that reflect the internal aspects of religiosity (i.e., believing and behaving). We do not observe a significant association for those dimensions that reflect more external aspects of religion (i.e., belonging and bonding). These results suggest that the social capital perspective prevails the value perspective, at least when internal aspects of religiosity are concerned. More generally, our study demonstrates the importance of distinguishing between different dimensions of religion when investigating the link between religion and entrepreneurship.

## Introduction

Extant research shows that entrepreneurship rates in developed countries vary according to economic development and various other determinants. However, country differences in entrepreneurship rates appear to be rather persistent over time (Freytag and Thurik 2007; Thurik and Dejardin 2012), and are thought to be related to elements of culture such as uncertainty avoidance (Wennekers et al. 2007). In this context, culture can be understood as “those customary beliefs and values that ethnic, religious, and social groups transmit fairly unchanged from generation to generation.” (Guiso et al. 2006, p.23). In the present paper, we investigate the relation between religion, as a concept related but distinct from culture, and the incidence of entrepreneurship. Religion and culture are related in the sense that the higher the numbers of religious individuals the more the norms, values and traditions are reflected in culture (Tomes 1985; Williamson 2000), and they are distinct in the sense that (relatively) small religious groups may deviate in terms of values and norms from mainstream culture.

Entrepreneurs and small businesses make positive contributions to economies in terms of innovation, employment generation, productivity, and growth (Carree and Thurik 2010; Van Praag and Versloot 2007). Consequently, the creation, growth, and survival of new ventures are viewed by policymakers as key elements of economic development (Audretsch 2007). Although no single universally accepted definition of entrepreneurship exists, two basic notions of entrepreneurship prevail: entrepreneurship as a *type of behavior* and entrepreneurship as an *occupation.* 1 The role of religious adherence in the occupational decision making of individuals to become an entrepreneur has recently received attention in the literature (Audretsch et al. 2013; Carswell and Rolland 2007; Dana 2009). This growing stream of literature is relevant for our understanding of economic development as occupational choices (e.g. entrepreneur versus employee) can both be enhanced and restricted by religion through for example religious practices and values in a society (Inglehart and Baker 2000; König et al. 2007; Norris and Inglehart 2004). However, quantitative country level studies such as the current study are scant (Rietveld and Van Burg 2014). One notable exception is a recent study by Zelekha et al. (2014). Using a cross-country dataset on business founders collected from LinkedIn, these authors find support for the thesis that various religions have different effects on entrepreneurial activity at the country level. In addition, the authors find that a country’s prevalence of entrepreneurship is primarily related to the majority religion rather than to the relative sizes of different religious groups. This finding appears to confirm earlier suggestions by Dana (2009) that the values of the majority religion have a macro-effect on entrepreneurial activity via institutional forces such as cultural values and norms.

Value differences between religious and non-religious groups (Saroglou et al. 2004; Schwartz and Huisman 1995), and across religious groups (Audretsch et al. 2013; Dana 2007, 2009) may influence the propensity to be an entrepreneur. As such, the composition of the population in terms of religious groups may be associated with the rate of entrepreneurship at the country level. The number of citizens adhering to a specific religion changes over time (Maoz and Henderson 2013; Norris and Inglehart 2004), and while some religions decline in a country, other religions enter and grow, for example, by the entrance of migrants. These changes in a population’s composition of religious groups may influence aggregate entrepreneurship rates, likely in the long term because the influence of value systems, including religious value systems, on institutions tends to change slowly (Inglehart and Baker 2000). Put differently, changes in the religious composition of the population are likely to influence the preferences and values that are operative in the selection process of what is accepted as appropriate (occupational) behavior (Nelson 2006).

The aim of this study is to increase our understanding of the complex relation between religion and entrepreneurship at the country level. In particular, we are interested in how religion influences the occupational choices of labor market participants at the aggregate level, i.e., a country’s labor force distribution between employees and business owners. Therefore, we use a measure of entrepreneurship consistent with occupational choice theory (e.g., Lucas 1978), namely, the business ownership rate, which expresses the number of business owners in a country as a share of the labor force. In our empirical analysis, we explain variation in the (non-agricultural) business ownership rate across OECD countries by different measures representing various dimensions of religion. Herewith, we conceive religion as a multidimensional construct (Hill 2005) where each dimension offers distinct information on how religion affects individual’s lives and decision-making processes (Saroglou 2011). In each regression, we correct for several country-level determinants of entrepreneurship as employed in earlier studies reflecting primarily cross-country differences attributed to economic circumstances (Freytag and Thurik 2007), such as GDP per capita, the share of services in total employment and labor productivity (e.g., Wennekers et al. 2007).

Our contribution is threefold. *First*, by providing systematic cross-country empirical evidence on the complex relation between religion and entrepreneurship, our study helps explaining the seemingly persistent country differences in entrepreneurship rates. A specific advantage of explaining entrepreneurship at the country level is that occupational choices of all labor force participants in a country are considered simultaneously, rather than an individual’s occupational choice being considered in isolation (even when also country level determinants are included, as in multilevel approaches). Specifically, in contrast to the individual level, the country level measurement of entrepreneurship is able to take industry structure (in terms of the distribution of small and large businesses) into account.^2^ Additionally, a cross-country study is warranted since religion is a collective phenomenon that transcends the individual that identifies with religious values (Nelson 2006; Williamson 2000). *Second*, whereas other studies refer to possible mechanisms but formulate expectations based on empirical insights, we specify mechanisms and expectations based on insights from theory. More specifically, we apply a value perspective and a social capital perspective to assess the motivational goals of religious individuals and self-employed individuals. *Third*, we distinguish between four universal dimensions of religion: *belonging* to a religious denomination, *believing* certain religious propositions, *bonding* by means of spiritual practices and rituals, and *behaving* according to values privileged by religion (Saroglou et al. 2004). We explore whether these dimensions influence the entrepreneurship rate at the country level differently. With each dimension representing different psychological processes, products, goals and outcomes (Saroglou 2011, p.1325), it seems reasonable to believe that different dimensions have different impacts on decision-making processes such as occupational choices. Moreover, in highly secularized societies public religious practice may be less relevant compared to personal or intrinsically motivated religiosity (Norris and Inglehart 2004). However, little is known about the impact of the different dimensions of religion.

The set-up of this paper is as follows. The next section provides a literature review about the relation between religion and entrepreneurship. Following sections cover our theoretical development, data and methods, empirical results, discussion and conclusions, respectively.

## Literature background

### Religion

For many people around the world, religious beliefs are central to their everyday lives, as religion provides the moral codes by which they live (Geertz 1993). Religious beliefs not only provide a framework of general ideas or systems of meaning but also shape these ideas and systems (Geertz 1993; Iannaccone 1998). In his influential contribution on the economics of religion, Iannoccone defines religion as “any shared set of beliefs, activities, and institutions premised upon faith in supernatural forces” (Iannaccone 1998, p. 1466). As such, religious beliefs are believed to be socially embedded in society and part of informal institutions with a persistent influence on the long-term character of that society’s economy (Norris and Inglehart 2004; Williamson 2000; North 1991).

Religion is a multidimensional concept that includes cognitive, emotional, behavioral, interpersonal, and physiological dimensions (Hill and Pargament 2003). As a result, earlier research has proposed a variety of definitions of religion that reflect the multidimensionality of the concept (Hill and Pargament 2003; Idler et al. 2003). The absence of a commonly used measure of religiousness has hampered research to date, resulting in inconsistent findings (Idler et al. 2003). We argue that investigating the influence of different dimensions separately is essential because these dimensions reflect “interconnected, but partly distinct, underlying psychological processes, religious products, goals, functions, and mechanisms explaining religion’s outcomes on individuals’ lives and society” (Saroglou 2011, p. 1334).

### Religion and economic behavior

Religion affects the economic behavior of individuals (Blum and Dudley 2001; Iannaccone 1998; Tomes 1985). Notably, Weber (1930) and other researchers argue that religious beliefs shape economic activities and that such beliefs played a critical role in the rise of the capitalist enterprise and industrialization of Western Europe. Other authors have emphasized the relationship between religion and the economic behavior of individuals (Hilary and Hui 2009; Kumar et al. 2011; Noussair et al. 2013). For example, Hilary and Hui (2009) and Noussair et al. (2013) demonstrate that higher levels of religiosity are associated with higher levels of risk aversion affecting managerial decision making, organizational behavior, and financial market outcomes.

Additionally, religion may also be related to an individual’s occupational choice. As religious groups are characterized by different values compared to non-religious groups (Rokeach 1969; Schwartz and Huisman 1995),^3^ individuals adhering to a religious denomination may differ in their likelihood of choosing the entrepreneurial option as a career choice. Furthermore, empirical evidence also appears to suggest that differences in religious denominations influence self-employment (Audretsch et al. 2013; Carswell and Rolland 2007; Dodd and Seaman 1998). For example, studying a sample of Indian individuals, Audretsch et al. (2013) find that adherents of Islam and Jainism have a higher propensity to be engaged in self-employment activities, whereas adherents of Hinduism and Buddhism have a lower propensity to be self-employed.

### Religion and prevalence of entrepreneurship

Affecting the economic behavior of individuals, religion may also be related to the prevalence of entrepreneurship (business ownership) at the country level. A limited but growing number of recent studies explore this idea (Table 1). Table 1 reveals the diversity of these studies in terms of key measures for both religiousness and entrepreneurship and a seemingly positive relationship between (Christian) religion and the prevalence of entrepreneurship. Additionally, we note that the formulation of hypotheses and expectations in these studies are more driven by empirical insights than by theoretical insights. Heterogeneity is observed in terms of measures used for religiousness and entrepreneurship. The entrepreneurship measures used include early-stage entrepreneurship (Galbraith and Galbraith 2007; Henley 2014), self-employment (Parboteeah et al. 2015) and entrepreneurial activity based on LinkedIn (Zelekha et al. 2014). The latter authors use the social network site LinkedIn to compile a dataset measuring self-proclaimed entrepreneurs in mid- and high-technology sectors. However, how this measure relates to more established measures of entrepreneurship remains unclear. For religiousness, some studies use a single dimension for religiousness, such as religious affiliation (Henley 2014; Zelekha et al. 2014) whereas Parboteeah et al. (2015) investigate three different dimensions of religion (i.e., cognitive, normative and regulative dimensions) separately, others combine dimensions into one measure (Galbraith and Galbraith 2007). In addition, the relative sizes of different religious groups are considered (Henley 2014) as well as a country’s religious majority (Zelekha et al. 2014).

**Table 1:** Overview of empirical studies relating religion to entrepreneurship at the country level

Authors	Dataset	Sample	Measure for religion	Measure for entrepreneurship	Results
Galbraith and Galbraith (2007)	Global Entrepreneurship Monitor 2005/World Value Survey (1999–2004)	23 countries that have a historical Christian tradition	Combined measure of *Believing* (population percentage for “Do you consider yourself a religious person?”), *behaving* (population’s percentage for “How important is God in your life?”) and *bonding* (population’s percentage for “How often do you attend religious services?”).	Total early-stage entrepreneurial activity and opportunity total early-stage entrepreneurial activity	Suggests a positive effect
Zelekha et al. (2014)	LinkedIn data October 2011/Source for religion variables not described	176 countries	*Belonging* (population’s percentage of affiliation, dummy variable for country’s majority religion) and *other* (religious diversity, not used in analysis).	Number of LinkedIn entrepreneurs in the mid- and high-technology sectors per 1 million population in a country	Different effects for different religious denominations
Henley (2014)	Global Entrepreneurship Monitor 2011-2012/Compendium of compiled data from international surveys 2010 (e.g., World Christian Database)	74 countries	*Belonging* (population’s percentage of affiliation) and other (Religious Pluralism Index (Herfindahl-Hirschman Index))	Total early-stage entrepreneurial activity, nascent entrepreneurship, necessity entrepreneurship, improvement-driven entrepreneurship	Positive effect in particular for new forms of Christianity (evangelical, charismatic and Pentecostal movement)
Parboteeah et al. (2015)	Flash Eurobarometer Survey No. 283 on Entrepreneurship 2010/World Value Survey.	27 (predominantly Christian) countries	*Believing* (five items representing the country percentage of people who believe in religion), *bonding* (the country percentage of people who pray and attend church at least once a week), and other (dummy for state religion)	Self-employment	Weakly positive significant result for *believing* and non-significant results for *bonding* and the presence of a state religion

It is frequently suggested that religion and particularly values associated with religion provide circumstances conducive to entrepreneurial activity (Dodd and Seaman 1998; Henley 2014; Parboteeah et al. 2015). In particular, the work of Weber (1930) is repeatedly cited in this line of reasoning. According to Weber, Protestant Christian values such as ambition, perseverance, and wealth accumulation serve as important motivators for the economic behavior of religious individuals. At the country level, these Protestant Christian values as well as values from other religions influence the context in which individuals make their decisions, providing a set of legitimate options that include engagement in entrepreneurial activity. These findings suggest the existence of a direct effect of religion on the behavior of society’s religious members and an indirect effect of religion as part of a country’s institutional context. The indirect effect implies that even if an individual is not religious, residing in a context influenced by the values propagated by religion may affect his or her entrepreneurial engagement (Dana 2009; Parboteeah et al. 2015; Zelekha et al. 2014).^4^

Next, we outline two theoretical perspectives that may provide further insight into the above-mentioned mechanisms: a value perspective and a social capital perspective. We use these theoretical perspectives to formulate expectations regarding the nature of the relation between religion and entrepreneurship. Although we take different dimensions of religiousness into account in our empirical analyses and, as mentioned before, it seems reasonable to believe that different dimensions relate differently to entrepreneurship, a lack of empirical insights and theory prevents us from formulating expectations for each dimension separately. This stresses the exploratory nature of this research.

## Theoretical development

### Values

Religions may promote or discourage entrepreneurial efforts through values. It is suggested that religions cultivate certain values while de-emphasizing others that may stimulate or hinder entrepreneurship (Dana and Dana 2008; Dana 2009; Zelekha et al. 2014). Values are understood as the criteria or goals used by individuals to evaluate others, to justify what is socially desirable and to assess and explain their actions (Rokeach 1969). Values are abstract and independent of specific actions and situations. We argue that if a society contains more religious people with specific values that may be conducive (unfavorable) to entrepreneurship, then higher (lower) levels of entrepreneurship will prevail at the country level.^5^

Several studies suggest that the value priorities of religious individuals differ from the priorities of those who are not religious (Roccas 2005; Rokeach 1969; Schwartz and Huisman 1995). An early study by Rokeach (1969), for example, reports that religious individuals (Christian oriented) consistently gave more priority to salvation than non-religious individuals did, whereas the non-religious gave more priority to pleasure and independence. Subsequent studies using different value theories and models suggest that religion is positively associated with values such as conformity to social expectations and preferences for the harmony and stability of society and family and negatively associated with self-direction, achievement and personal success (summarized in Schwartz and Huisman 1995).

Moreover, for entrepreneurship, it has been suggested that those who are self-employed have different value priorities compared with non-self-employed individuals (Beugelsdijk and Noorderhaven 2005; Noseleit 2010). However, the use of different value models across the above-mentioned studies hinders comparisons of the value priorities found for religious individuals with those of entrepreneurs. We draw on studies that share a common value typology to compare the values of religious individuals and entrepreneurs: Schwartz’s Theory of Values (Schwartz and Huisman 1995). Schwartz was the first to propose a theory of the content and structure of values (Schwartz 1992). As opposed to previous value typologies established in psychology (e.g. Rokeach 1969) Schwartz’s Value Theory organizes a range of values in a cross-culturally stable hierarchical model (Roccas 2005). The model includes a set of 10 motivationally distinct types of values including power, achievement, hedonism, stimulation, self-direction, universalism, benevolence, tradition, conformity, and security (for a complete description, see Schwartz 1992). These values vary in importance across individuals, are trans-situational and above all, serve as guiding principles in people’s lives (Schwartz 1992). Moreover, the theory stipulates relations among values in terms of conflict and compatibility. More specifically, action undertaken on the basis of certain values (e.g. stimulation derived from novelty and experiencing excitement) may conflict with the pursuit of other values (e.g. security values) (Roccas 2005).

Schwartz’s values were first studied in relationship to religiosity by Schwartz and Huisman in 1995. A multitude of studies on the relationship of religiosity and Schwartz values followed. Based on a meta-analysis of 21 samples across 15 countries using Schwartz typology, Saroglou et al. (2004) conclude that those who are religious attribute high importance to values that promote the status quo of social and individual order and relatively low importance to values that endorse openness to change, independence in thought and action, and self-enhancement and hedonism. These patterns of correlation between religious individuals and their values were consistent and constant across different religious denominations and cultures (Roccas 2005; Saroglou et al. 2004). At the aggregate level, the values considered most important appear to be related to a country’s dominant religion (Inglehart and Baker 2000). For example, countries with a Protestant religious majority attribute high importance to both self-expression and secular-rational values, whereas countries with a Catholic majority attribute a moderate level of importance to these types of values (Inglehart and Baker 2000; Norris and Inglehart 2004).

With respect to entrepreneurship, Licht (2007) uses Swartz’s typology and argues that openness to change, expressed as seeking novelty and challenges in life and being independent in thought, is the distinguishing characteristic of self-employed individuals. Thus, entrepreneurs assign lower priority to opposing values, such as security, conformity, and tradition values. Noseleit (2010) empirically explores the hypotheses posed by Licht (2007) using a cross-country dataset of self-employed individuals in Western Europe. Consistent with Licht’s hypotheses, self-employed individuals assign high priority to values emphasizing openness to change and self-enhancement and consider conservation to be less important.

The combination of insights of Saroglou et al. (2004), Licht (2007) and Noseleit (2010) suggests opposing motivational goals for religious individuals and self-employed individuals. Religious individuals assign high importance to values that endorse conservation (i.e., the stability of society, social order, restraint in actions, and respect and acceptance of tradition). Self-employed individuals, on the contrary, attribute high importance to openness to change and personal success and ambition. Drawing on the aggregate psychological characteristics approach (Davidsson 1995; Freytag and Thurik 2007), we postulate that the value priorities of religious individuals in a country affect a country’s level of entrepreneurship. Given the opposing value differences between religious individuals and those values deemed important to entrepreneurs, based on Schwartz’s Value Theory we may expect a negative relationship between religion and entrepreneurship at the country level.

### Social capital

Religion may also be related to entrepreneurial activity through the development of social capital. Being religious and belonging to a religious group may serve as a source of social capital. The literature relating social capital and entrepreneurship is well developed (Brüderl and Preisendörfer 1998; Davidsson and Honig 2003). Social capital refers to an individual’s capacity to obtain benefits from his or her social structures, personal network and relationships (Davidsson and Honig 2003). Whether we conceive of entrepreneurs as making judgmental decisions regarding the coordination of scarce resources (Casson 1982), introducing new and innovative products and services to the market (Schumpeter 1934) or serving as arbitrageurs alert to profitable opportunities (Kirzner 1973), social networking and embeddedness in a social context are central to the very idea of entrepreneurship. New business creation as well as growing and sustaining an existing business requires exploiting, maintaining and extending one’s social relationships and network (Brüderl and Preisendörfer 1998). Social capital is positively associated with entrepreneurship, as it provides access to information regarding opportunities and stakeholders such as customers and competitors, eases access to financial and human resources, creates admission to markets, and potentially creates legitimacy from entrepreneurship (Abell et al. 2001).

According to Parker (2009), the general consensus is that membership in networks such as trade associations and churches enhance entrepreneurs’ performance and survival. However, some negative consequences of social capital have also been suggested, including the exclusion of outsiders, excessive claims on group members, and restriction on individual freedom (Portes 2000), which may restrict the scope for expanding business operations into broader markets (Parker 2009). Religion serves as a source of social capital. Putnam (2000), for example, argues that faith communities can be perceived as the most important source of social capital in American society. In addition, Fukuyama (2001) claims that values encouraged by religious communities, such as honesty and reliability, are conducive to better business relationships. As such, social capital is a byproduct of religious affiliation when members address one another in an economic context.

Based on these social capital arguments, we expect a positive relationship between religion and entrepreneurial activity at the country level. In addition, the maintenance of one’s social network and the strong focus on group members may limit the scope for expanding operations beyond one’s religious group and may result in a relatively high number of business owners running smaller businesses on average (i.e., a high business ownership rate). Hence, based on the social capital perspective, we may expect a positive relationship between religion and entrepreneurship at the country level.

## Data & method

This section describes the empirical analysis method used in this study. It is important to note that we perform a country level study. All measures are derived at the country level. Our sample consists of 30 countries that are available in the different data sources that we use and that belong to the Organization for Economic Co-operation and Development (OECD): Australia, Austria, Belgium, Canada, the Czech Republic, Denmark, Finland, France, Germany, Greece, Hungary, Iceland, Ireland, Italy, Japan, Korea, Luxembourg, Mexico, New Zealand, Norway, Poland, Portugal, Slovak Republic, Spain, Sweden, Switzerland, the Netherlands, Turkey, the United States of America, and the United Kingdom.

### Dependent variable: business ownership rate

In this paper, we adhere to the occupational notion of entrepreneurship and use a country’s business ownership rate (the share of the labor force that is a business owner as primary occupation) as a measure of the level of entrepreneurship. More specifically, the dependent variable in our analyses is the harmonized (non-agricultural) business ownership rate, defined as the number of unincorporated and incorporated self-employed (excluding agriculture) as a fraction of the labor force.^6^ The data source is the Compendia database (Van Stel 2005), originally composed by Panteia/EIM.^7^ In this database, self-employment numbers as published in *OECD Labour Force Statistics* are corrected for measurement differences across countries and over time and thus harmonized.^8^ This variable is available for each year for the 1972–2012 period.

### Independent variables: dimensions of religion

Following Saroglou (2011), we distinguish between four dimensions of religion representing different psychological processes: belonging, believing, bonding, and behaving. This distinction is particularly useful in the context of this study, as these dimensions are assumed to be universally present albeit different across religions and cultural contexts in terms of their intensity and modes of expression (Saroglou 2011). Although distinguishing between dimensions is not new (Glock 1962; Tarakeshwar et al. 2003; Verbit 1970) Saroglou’s Big Four Religious Dimensions integrates and extends previous work, offers a meaningful organization to the variation of religious forms and is fit to cross-country research. *Belonging* refers to belonging to a religious community or (transhistorical) group with a common history and future. This dimension relates to social processes of in-group identification serving as a reference point for shared norms and what is considered socially desirable. *Believing* is considered a basic universal aspect of religion. Contrary to belonging, believing concerns a cognitive function of religion and refers to external transcendence expressed by most religions by the existence of one or several gods. Belief in some form of transcendence is related to ideas regarding what is considered “truth” and processes of giving meaning. *Bonding* refers to “self-transcendent experiences that bond the individual with what it perceives to be the transcendent ‘reality’ with others, and/or with the inner-self” (Saroglou 2011, p. 1326). Bonding can be expressed as participating in public rituals such as church attendance and/or private spiritual practices such as prayer and meditation. *Behaving* refers to the behavior of an individual according to the norms and moral standards associated with one’s religious convictions. In sum, the four dimensions of belonging, believing, bonding, and behaving represent the social, cognitive, emotional, and moral elements of religion, respectively (Saroglou 2011).

Our four main independent variables are measures of the *belonging*, *believing*, *bonding*, and *behaving* dimensions of religion. All measures are obtained from the *European Value Survey* (EVS 2011). For *belonging*, we use the responses to the question ‘Do you belong to a religious denomination?’ (yes/no). For *believing*, the question ‘Do you believe in God?’ (yes/no) is used. For *bonding*, answers to the question ‘Do you take time for prayer/meditation’ (yes/no) are used. Finally, for *behaving*, we use the subjective evaluation of the importance of God in someone’s life (“How important is God in your life?”). Answer categories range from 1 indicating “not at all” to 10 indicating “very important”. The mean values per country are divided by 10 to rescale this variable to a value between 0 and 1. We derive the religion measures from the individual-level responses by averaging the responses at the country level. If a country-wave observation was not available in the EVS, we took it from the *World Value Survey* if available there (Inglehart et al. 1998). The four waves of data collection in EVS are for the 1981–1984, 1990–1993, 1999–2001, and 2008–2010 periods. We selected waves 1 (1981–1984), 2 (1989–1993), 4 (1999–2004), and 5 (2005–2007) from the WVS as comparable times of data collection for imputation.

### Control variables

Our main control variable is the level of economic development as measured by *real per capita income* (source: OECD National Accounts). It is well documented that self-employment rates depend on several mechanisms related to the level of economic development. These mechanisms include the transition from an agricultural economy to an economy dominated by manufacturing and subsequently to a service economy. Other mechanisms include the rise in real wages relative to entrepreneurial income (Lucas 1978) and the varying importance of scale economies based on the level of economic development. For an overview, see Wennekers et al. (2010). Overall for the OECD countries in our sample, we expect a negative association between per capita income and business ownership.

We also include the *female share in the labor force* (source: OECD Labour Force Statistics). As business ownership rates tend to be lower for females than for males, a higher share of women in the labor force is expected to be negatively related to business ownership at the country level. The *employment share of services in the economy* (source: OECD National Accounts) is expected to be positively related to the economy-wide business ownership rate, as self-employment rates are higher in services than in other more capital-intensive sectors of economic activity. The sign of *labor productivity* (source: OECD National Accounts) is indeterminate from theory. On the one hand, high levels of productivity raise competitiveness and potential profits, making entrepreneurship an attractive option. On the other hand, high productivity levels often translate into high wages, making wage-work relatively attractive. The expected sign of *population density* (source: OECD Labour Force Statistics and Grote Winkler Prins encyclopedia) is positive because of the high local demand and agglomeration advantages, making entrepreneurship an attractive option in densely populated areas. *Tax revenues as a percentage of GDP* (source: OECD Revenue Statistics) is expected to be negatively related to business ownership, as a high-tax environment decreases the appeal of operating a business. The association between *R&D as a percentage of GDP* (source: OECD Science, Technology and R&D indicators) and business ownership is expected to be negative because R&D activity is dominated by large firms (Cohen and Klepper 1992; Sørensen and Stuart 2000). The *gross replacement rate* (GRR; source OECD Benefits and Wages data base) is expected to be negatively related to business ownership, as higher social security benefits typically favor wage-earning employees and not self-employed individuals. Higher GRRs thus make self-employment a less attractive labor market option. Higher *tertiary education rates* (source: World Bank EdStats data base) are associated with more successful entrepreneurs who run larger businesses on average. The number of business owners is therefore expected to be lower in such environments (Van Praag and Van Stel 2013). The expected sign of the *harmonized unemployment rate* (HUR; source: OECD Main Economic Indicators) is indeterminate from theory. On the one hand, high levels of unemployment may lead to more necessity entrepreneurship as a result of unemployment. On the other hand, in high-unemployment environments, demand for products and services may be lower, making entrepreneurship less attractive (Thurik et al. 2008). Finally, *the share of individuals in the 25–39 age category* (source: US Census Bureau) may positively influence business ownership, as self-employment rates tend to be higher in this category. For more information regarding these control variables and how they influence the business ownership rate at the country level, we refer to Wennekers et al. (2007). Missing values for the controls variables were imputed by linearly interpolating variables (when earlier and later observations were available, as in Wennekers et al. 2007).

### Empirical model

We test for an association between religion and entrepreneurship at the country level. Because a country’s composition of religious groups tends to change slowly over time (Maoz and Henderson 2013) and because country differences in business ownership tend to be rather stable over time (Freytag and Thurik 2007), we focus on explaining variations across countries rather than explaining variations over time.^9^ Accordingly, we use a pooled OLS estimator to explain the (non-agricultural) business ownership rate from our four different measures for religion (rather than a fixed effects estimator). As the *European Value Survey* was conducted in 1984, 1993, 2001, and 2010 (the end years of data collection), we selected these years for our analyses. Thus, the observations included for each country are approximately 9 years apart. We assume that these intervals are sufficiently long to consider the country samples for these years sufficiently independent to warrant pooling them in one regression. Nevertheless, we also cluster standard errors by country. Descriptive statistics of the dependent and independent variables in our sample are presented in Table 2.

**Table 2:** Descriptive statistics for dependent and independent variables included in the empirical analysis

	Year			
	1984	1993	2001	2010
Business ownership rate	0.10 (0.04)	0.11 (0.04)	0.11 (0.04)	0.11 (0.04)
*N* (countries)	25	30	30	30
Belonging (0–1)	0.87 (0.12)	0.75 (0.17)	0.76 (0.17)	0.73 (0.18)
*N* (countries)	20	25	26	23
Believing (0–1)	0.78 (0.14)	0.72 (0.22)	0.75 (0.22)	0.72 (0.17)
*N* (countries)	19	23	25	24
Bonding (0–1)	0.60 (0.16)	0.60 (0.18)	0.65 (0.17)	0.62 (0.16)
*N* (countries)	14	23	26	30
Behaving (0–1)	0.60 (0.14)	0.60 (0.15)	0.62 (0.17)	0.60 (0.16)
*N* (countries)	19	25	26	30

Standard deviations between brackets

Our empirical strategy resembles that of Wennekers et al. (2007). In each regression, we include GDP per capita and year dummies as our main control variables. In addition, we iteratively include the other control variables described in Section 3.3. In a final model, we include all variables that are found to be significant at the 10 % level when they are added individually to the base model (which includes a measure of religion plus the main control variables). The empirical results will shed light on which mechanism relating religion to entrepreneurship (values or social capital, see the *Theoretical Development* section) prevails at the country level.

## Empirical results

The descriptive statistics of our sample (Table 2) show that the average business ownership rate is rather stable over time. The rate increases from 10 to 11 % from 1984 to 2010. However, the development of this country average masks considerable variation in the business ownership rate across countries and over time. Where the percentage of individuals belonging to a religious denomination clearly decreases over time (from 87 % to 73 %), the percentages for the believing, bonding, and behaving dimensions of religion are relatively stable over the period from 1984 to 2010.^10^ The correlation between the business ownership rate and religion is significantly positive for *believing*, *bonding*, and *behaving*. For *belonging*, the correlation is not significant (Table 3).

**Table 3:** Correlation table of dependent and independent variables included in the empirical analysis

	Business ownership rate	Belonging	Believing	Bonding	Behaving
Business ownership rate	1.00				
*N*	115				
Belonging	−0.03	1.00			
*N*	92	94			
Believing	0.36***	0.52***	1.00		
*N*	89	90	91		
Bonding	0.30**	0.45***	0.64***	1.00	
*N*	93	86	86	93	
Behaving	0.42***	0.53***	0.75***	0.86***	1.00
*N*	98	92	90	93	100

* *p* < 0.05, ** *p* < 0.01, *** *p* < 0.001

Table 4 reports the results for the final models resulting from the iterative procedure described in the *Data & Method* section (for intermediate results, see Tables 5, 6, 7 and 8 in the appendix; note that only the significant control variables from Tables 5, 6, 7 and 8, i.e. the final columns from these tables, come back in our main Table 4).^11^ These models explain the business ownership rate using the four dimensions of religion.^12^ For *belonging* (Model 1), the final model includes the control variables GDP per capita, female labor share, tax revenues, and gross replacement rate. All these variables have a negative coefficient, as expected, but only GDP per capita is significant in the final model. In addition, the coefficient for *belonging* is not statistically significant. The final model does not include observations for New Zealand (because no measure for belonging is available for this country in our sample) or Mexico (because the gross replacement rate is not available for this country). The regression coefficient for *belonging* is also insignificant in a model that includes only GDP per capita and year dummies as control variables.

**Table 4:** The association between religion and the business ownership rate

	(1)	(2)	(3)	(4)
Belonging	−0.002			
	(0.032)			
Believing		0.059**		
		(0.025)		
Bonding			0.020	
			(0.043)	
Behaving				0.095**
				(0.044)
GDP per capita	−0.024*	−0.026*	−0.035**	−0.032***
	(0.014)	(0.015)	(0.013)	(0.011)
Female labor share	−0.301		−0.312	
	(0.248)		(0.227)	
Share services				
Labor productivity				
Population density				0.072*
				(0.041)
Tax revenues	−0.046		−0.046	
	(0.136)		(0.152)	
Research & development		−1.160**		
		(0.553)		
Gross replacement rate	−0.041	−0.035	−0.031	−0.043
	(0.049)	(0.029)	(0.045)	(0.029)
Tertiary education				
Harmonized unemployment rate		−0.286**	−0.223*	−0.141
		(0.107)	(0.124)	(0.123)
Share of 25–39 age group in adult population	0.015			
	(0.175)			
Year dummy 1993	0.017	0.014**	0.024**	0.008
	(0.011)	(0.006)	(0.010)	(0.007)
Year dummy 2001	0.031**	0.012	0.032**	0.014
	(0.015)	(0.009)	(0.012)	(0.010)
Year dummy 2010	0.035*	0.022**	0.042**	0.022**
	(0.019)	(0.010)	(0.017)	(0.010)
Constant	0.266**	0.137***	0.289***	0.091**
	(0.111)	(0.027)	(0.091)	(0.034)
*N*	81	75	83	87
*N* (countries)	28	26	29	29
*R* ^2^	0.310	0.344	0.352	0.330

Dependent variable is the (non-agricultural) business ownership rate. Results are from a pooled sample estimation for 1984 (reference year), 1993, 2001 and 2010. Standard errors are reported between brackets and clustered by country

* *p* < 0.10, ** *p* < 0.05, *** *p* < 0.01

Model 2 includes the *believing* dimension of religion. The regression coefficient for *believing* is significantly positive at the 5% level, indicating that higher levels of *believing* are associated with higher levels of business ownership in a country. The coefficient of GDP per capita is also significant in this model and shows the expected negative sign. The coefficients for R&D and the gross replacement rate are both in the expected negative direction. The negative regression coefficient for the harmonized unemployment rate suggests that in high-unemployment environments, entrepreneurship is less attractive because of the lower demand for products and services. The results of the final model in Model 2 are based on 26 countries, because R&D is not available for Korea, New Zealand and Switzerland, and the GRR for Korea and Mexico in the years in which *believing* is available.

For *bonding*, we find in Model 3 that *bonding* is not significantly associated with the business ownership rate (*p* = 0.64). GDP per capita, female labor share, tax revenues, the gross replace rate and the harmonized unemployment are included as control variables in the final model. All signs of the regression coefficients for these variables are in the expected direction. Mexico is not included in the final model sample, for the same reason as in Model 1.

Finally, Model 4 shows the regression results for the *behaving* dimension of religion. Behaving is positively associated with the business ownership rate in this model (*p* = 0.042). The signs of the regression coefficients for the control variables are in the expected direction, and significant for GDP per capita, population density, gross replacement rate and the harmonized unemployment rate. Again, Mexico is not included in the final model sample.

The Variance Inflation Factors of the variables in the four final models do not indicate problems with multicollinearity (all below 3.5). As a robustness check on the possible influence of missing observations on the regression results, we imputed the country mean (per year) for each missing value of each variable. Subsequently, we reran the four final models (with 30 × 4 = 120 observations each now). The regression coefficients we obtained for the four religion dimensions are similar in sign, magnitude and significance as those in the main analysis. In addition, as a robustness check on the possible influence of outliers, we determined the observations with an absolute standardized residual larger than 2 in the four final models and reran the regressions without these observations. The regression coefficients we obtained for the four religion dimensions are also qualitatively similar to those in the main analysis. Finally, to address the relatedness between religion and culture we include Hofstede’s well-known cultural dimensions (Hofstede et al. 2010) in the four final models. The inclusion of Hofstede’s dimensions does not change the regression coefficients for the religion dimensions qualitatively (see Table 9).

## Discussion

The empirical results show a significantly positive association between behaving and believing on the one hand, and a country’s business ownership rate on the other hand. In the case of belonging and bonding, we do not observe a significant association. A positive relationship between religion and the business ownership rate is revealed for those dimensions that reflect the internal aspects of religiosity, i.e., believing in God and the importance of God in one’s life. We do not observe a relationship for those dimensions that reflect an external manifestation of religion, i.e., affiliation and frequency of practice. With regard to this latter observation, it has been suggested that in highly secularized societies, as is the case for the majority of countries in our sample, the external manifestation of religion is less pronounced, whereas being religious is more reflected as an intrinsic orientation (Hodge 2003; Inglehart and Baker 2000; Saroglou 2011). More specifically, many individuals in the analyzed countries formally belong to a specific religion (belonging); however, their affiliation does not necessarily provide guidelines for everyday life and motivational goals. On the contrary, believing in God or some type of transcendence (believing) and related norms and moral standards reflected in one’s behavior (behaving) provide guidelines for what is socially (un)desirable and may thus influence actual behavior in everyday life, including occupational choice. These processes may or may not occur in organized contexts but are likely to be reflected at different levels of abstraction, including the country level. These findings may be in line with the view that values are typically determined early in life and tend to endure over time (Barnouw 1985; Hofstede et al. 2010; Inglehart and Baker 2000) whereas external manifestations of religion such as frequency of practice may be more prone to change. In this respect, a fruitful path for future research complementing the current study may include intergenerational shifts in values that may take place. Rather than country level data, studying the values that parents transmit to their children and its effects later in life with respect to occupational choices requires panel data at the individual level.

Interestingly, our findings are consistent with the study of Parboteeah et al. (2015): using a sample of predominantly Christian countries, they find that believing in God is positively related to a country’s rate of self-employment, but they do not find evidence for more external manifested proxies for religion (i.e., the frequency of church attendance and the presence of state religion). Potential explanations may be found in psychological research on religion and dogmatism that is, unjustified certainty regarding a set of principles laid down by an authority even in the face of disconfirming evidence (Roccas 2005; Saroglou 2002). In particular, values associated with religious dogmatism or “classic religiosity” (primarily the belonging dimension), such as high levels of preference for order and predictability, discomfort with ambiguity, and close-mindedness (Saroglou 2002), are more in conflict with entrepreneurial values than religious aspects not directly related to religious dogmas (i.e. emotionality, spirituality, quest of meaning and values). Nevertheless, our findings indicate that studies on the relationship between religion and entrepreneurship should carefully select the levels of measurement and provide justification for analyzing the influence of the distinct dimensions of religion separately.

Regarding the relative importance of the value versus social capital perspective, at first glance, the positive coefficient suggests that at the country level, the social capital mechanism prevails over the value mechanism. The findings appear to be consistent with the interpretation that countries with high levels of social capital (e.g., countries scoring high in the religion dimensions) are the countries with the highest business ownership rates. At the same time, as a high business ownership rate is associated with a lower average firm size, countries that are strong in the believing and behaving dimensions also have a lower average firm size, suggesting that religious people are strong in terms of establishing businesses but have a lower tendency to expand these businesses. On the one hand, this phenomenon may relate to the “protected market hypothesis” (Aldrich et al. 1985; Aldrich and Waldinger 1990), which postulates that when an initial market for a business remains limited to a specific community, such as a religious-ethnic group, its potential for growth is limited. On the other hand, the opposing values of religious individuals and values associated with entrepreneurship may also limit the willingness to grow a business and the drive for personal success and ambition, in line with Schwartz’s Value Theory, and resulting in smaller scale types of entrepreneurial activity. As such, a fruitful path for future research is to include other measures of entrepreneurship which emphasize different values, such as measures for ambitious entrepreneurship and high-growth entrepreneurship, and to relate them to religion.

A limitation of our data sample of OECD countries is that our results are not necessarily valid outside the context of these countries. Specifically, most countries in our sample are developed countries which are dominated by Christianity. Hence, our results may not be generalized to developing countries or countries dominated by other religions. Moreover, Western countries are special in the sense that a decreasing number of individuals belongs formally to a religious group, whereas the reverse pattern is true for the rest of the world (Norris and Inglehart 2004; Maoz and Henderson 2013). Another limitation concerns our relatively small sample of around 90 data points from 30 OECD countries. One may argue that the small number of significant relationships revealed might be the result of the sample size because small effects could not be detected. However, the inclusion of multiple observations per country helped to increase the power of the statistical analysis. Moreover, as regards our religion variables, we consider it unlikely that our indicators of belonging and bonding are insignificant due to the low number of observations, as absolute t-values are extremely low (below 0.5).

We also emphasize that the time dimension of the relationship between religion and entrepreneurship can more deeply be analyzed if more data over longer time periods become available. Greater data availability could help to shed light on possible causal relationships between these two phenomena, whereas our study provides insight into the cross-country association between religion and entrepreneurship. Nevertheless, under certain conditions, it is still possible to make statements regarding developments over time based on our regression estimates. In particular, due to our cross-country estimation, developments over time (e.g., in levels of economic development, religion, or business ownership) need to be interpreted *relative* to other countries. For instance, if a country receives more migrants than other countries, its religious composition might change relative to other countries (thereby affecting cross-country variation in the religion dimensions). In this context of country developments in deviation from other countries, our estimates may be used for explaining developments of the business ownership rate, in spite of the estimations being cross-sectional in nature.

Finally, we acknowledge that although studying the relation at the country level has certain advantages (as outlined in the Introduction), there are disadvantages as well. For instance, by aggregating data, meaningful variance across individuals within a country is lost (Hofmann 1997). Applying multilevel approaches is therefore also a promising route for future research in this field.

## Conclusion

Our systematic cross-country analysis contributes to our understanding of the complex relation between religion and entrepreneurship at the country level. In this regard, at least three complexities may be distinguished. *First*, the use of four different measures of religion that differentiate between four dimensions of religion (belonging, believing, bonding, and behaving) provides a broader view of this topic than earlier studies. The result indicating that only certain aspects of religion (i.e. intrinsic religious aspects) are associated with the business ownership rate contributes to our understanding of the persistent differences in entrepreneurship rates that exist between countries (Freytag and Thurik 2007).

*Second*, the relation between religion and entrepreneurship is also complex because different explanations are at play. In particular, as explained in Section 3, the values perspective emphasizes that the values that are important for religious individuals differ from those deemed important by entrepreneurs, thereby predicting a negative relation between religion and entrepreneurship. In contrast, the social capital perspective emphasizes that being strongly embedded in a social context such as a religious community provides important benefits for entrepreneurship, in particular benefits related to having a strong network. Hence, the social capital perspective predicts a positive relation. Our empirical results suggest that the social capital arguments dominate, at least when internal aspects of religiosity are concerned.

*Third*, although we have been explaining cross-country differences, under certain conditions outlined in the Discussion section, our estimates allow us to speculate about developments of religion and entrepreneurship at the country level in light of ongoing economic development. Inglehart and Baker (2000), for example, observe that despite a decline in participation in organized religion, religious beliefs persist, and spirituality (i.e., internal aspect related to religiosity) becomes more widespread when countries reach more advanced stages of economic development. Assuming these observations of Inglehart and Baker are correct, it would imply that our estimated negative impact of economic development (GDP per capita) on the business ownership rate, would be partly offset by an increasing part of the population that believes and behaves according to religious values, as we found that these internal aspects of religiosity are positively related to business ownership.

The recent publication of several studies on the relationship between religion and entrepreneurship indicates the interest of the scientific community in this topic. Our study shows that it is relevant to consider which dimension of religion is used in this type of research. We conclude that religion is associated with the business ownership rate via the internal aspects of religiosity, i.e., believing in God and the importance of God in one’s life, rather than through external manifestations of religion, i.e., affiliation and frequency of religious practice. The results in this paper indicate that research in this direction is clearly warranted.

## Appendix

Table 5

**Table 5:** The association between religion (Belonging) and the business ownership rate

	Baseline	(1)	(2)	(3)	(4)	(5)	(6)	(7)	(8)	(9)	(10)	Final
Belonging	−0.004	−0.014	−0.007	−0.008	0.031	−0.011	−0.014	0.006	−0.013	0.004	−0.022	−0.002
	(0.029)	(0.027)	(0.029)	(0.029)	(0.041)	(0.027)	(0.023)	(0.030)	(0.029)	(0.028)	(0.034)	(0.032)
GDP per capita	−0.037**	−0.028**	−0.036**	−0.082**	−0.037**	−0.028**	−0.022	−0.030**	−0.037**	−0.048***	−0.029**	−0.024*
	(0.014)	(0.013)	(0.016)	(0.034)	(0.013)	(0.011)	(0.017)	(0.011)	(0.016)	(0.013)	(0.012)	(0.014)
Female labor share		−0.359**										−0.301
		(0.175)										(0.248)
Share services			−0.025									
			(0.076)									
Labor productivity				0.143								
				(0.122)								
Population density					0.088							
					(0.059)							
Tax revenues						−0.205***						−0.046
						(0.070)						(0.136)
Research & development							−1.140					
							(0.718)					
Gross replacement rate								−0.069**				−0.041
								(0.032)				(0.049)
Tertiary education									0.029			
									(0.040)			
Harmonized unemployment rate										−0.206		
										(0.126)		
Share of 25–39 age group in adult population											0.288*	0.015
											(0.164)	(0.175)
Year dummy 1993	0.002	0.014	0.006	0.005	0.007	0.007	0.004	0.009	−0.003	0.008	0.003	0.017
	(0.009)	(0.009)	(0.009)	(0.008)	(0.010)	(0.007)	(0.009)	(0.008)	(0.012)	(0.008)	(0.008)	(0.011)
Year dummy 2001	0.021*	0.029**	0.022*	0.021*	0.025*	0.020*	0.021*	0.019	0.013	0.022**	0.027**	0.031**
	(0.011)	(0.013)	(0.012)	(0.011)	(0.012)	(0.010)	(0.012)	(0.012)	(0.016)	(0.010)	(0.011)	(0.015)
Year dummy 2010	0.021*	0.034**	0.023*	0.019	0.026*	0.020*	0.018	0.021*	0.006	0.028**	0.035**	0.035*
	(0.012)	(0.016)	(0.013)	(0.012)	(0.013)	(0.011)	(0.011)	(0.011)	(0.021)	(0.012)	(0.014)	(0.019)
Constant	0.141***	0.284***	0.158***	0.124***	0.098**	0.210***	0.151***	0.143***	0.143***	0.162***	0.018	0.266**
	(0.028)	(0.071)	(0.044)	(0.040)	(0.039)	(0.034)	(0.023)	(0.024)	(0.027)	(0.031)	(0.063)	(0.111)
*N*	92	91	91	91	92	91	88	81	85	91	92	81
*R* ^2^	0.16	0.32	0.18	0.23	0.21	0.32	0.18	0.20	0.13	0.22	0.23	0.31

Dependent variable is the (non-agricultural) business ownership rate. Results are from a pooled sample estimation for 1984 (reference year), 1993, 2001 and 2010. Standard errors are reported between brackets and clustered by country

* *p* < 0.10, ** *p* < 0.05, *** *p* < 0.01

Table 6

**Table 6:** The association between religion (Believing) and the business ownership rate

	Baseline	(1)	(2)	(3)	(4)	(5)	(6)	(7)	(8)	(9)	(10)	Final
Believing	0.058*	0.038	0.057*	0.051*	0.066*	0.034	0.047*	0.058*	0.056*	0.073**	0.049	0.059**
	(0.030)	(0.023)	(0.028)	(0.027)	(0.033)	(0.040)	(0.024)	(0.030)	(0.032)	(0.028)	(0.037)	(0.025)
GDP per capita	−0.027**	−0.022	−0.027*	−0.072**	−0.027*	−0.024**	−0.012	−0.022*	−0.021	−0.039***	−0.025*	−0.026*
	(0.013)	(0.013)	(0.015)	(0.028)	(0.013)	(0.011)	(0.018)	(0.012)	(0.016)	(0.011)	(0.012)	(0.015)
Female labor share		−0.297										
		(0.176)										
Share services			−0.016									
			(0.072)									
Labor productivity				0.144								
				(0.108)								
Population density					0.053							
					(0.035)							
Tax revenues						−0.145						
						(0.096)						
Research & development							−1.052*					−1.160**
							(0.600)					(0.553)
Gross replacement rate								−0.053*				−0.035
								(0.030)				(0.029)
Tertiary education									−0.006			
									(0.031)			
Harmonized unemployment rate										−0.264**		−0.286**
										(0.106)		(0.107)
Share of 25–39 age group in adult population											0.128	
											(0.172)	
Year dummy 1993	0.002	0.014	0.005	0.004	0.003	0.007	0.005	0.011	−0.001	0.008	0.003	0.014**
	(0.009)	(0.009)	(0.009)	(0.009)	(0.009)	(0.007)	(0.008)	(0.007)	(0.011)	(0.008)	(0.009)	(0.006)
Year dummy 2001	0.015	0.024**	0.016	0.016	0.017	0.016	0.017	0.014	0.013	0.015	0.019	0.012
	(0.011)	(0.012)	(0.011)	(0.011)	(0.011)	(0.010)	(0.010)	(0.011)	(0.014)	(0.010)	(0.012)	(0.009)
Year dummy 2010	0.019	0.032**	0.021	0.018	0.020*	0.019*	0.019*	0.019*	0.016	0.026**	0.026*	0.022**
	(0.011)	(0.014)	(0.013)	(0.012)	(0.011)	(0.010)	(0.011)	(0.010)	(0.017)	(0.011)	(0.015)	(0.010)
Constant	0.085***	0.213***	0.096**	0.069*	0.071**	0.151**	0.093***	0.092***	0.086***	0.107***	0.031	0.137***
	(0.026)	(0.072)	(0.037)	(0.036)	(0.031)	(0.058)	(0.023)	(0.027)	(0.028)	(0.024)	(0.064)	(0.027)
*N*	89	88	88	88	89	88	84	79	82	88	89	75
*R* ^2^	0.22	0.34	0.24	0.29	0.24	0.30	0.25	0.26	0.18	0.31	0.23	0.34

Dependent variable is the (non-agricultural) business ownership rate. Results are from a pooled sample estimation for 1984 (reference year), 1993, 2001 and 2010. Standard errors are reported between brackets and clustered by country

* *p* < 0.10, ** *p* < 0.05, *** *p* < 0.01

Table 7

**Table 7:** The association between religion (Bonding) and the business ownership rate

	Baseline	(1)	(2)	(3)	(4)	(5)	(6)	(7)	(8)	(9)	(10)	Final
Bonding	0.053	0.018	0.053*	0.044	0.064*	0.021	0.036	0.042	0.046	0.059*	0.033	0.020
	(0.034)	(0.030)	(0.031)	(0.033)	(0.036)	(0.046)	(0.028)	(0.033)	(0.039)	(0.032)	(0.046)	(0.043)
GDP per capita	−0.032**	−0.025*	−0.038**	−0.071**	−0.032**	−0.028**	−0.021	−0.028**	−0.031*	−0.044***	−0.028**	−0.035**
	(0.014)	(0.013)	(0.016)	(0.030)	(0.014)	(0.011)	(0.017)	(0.012)	(0.017)	(0.012)	(0.012)	(0.013)
Female labor share		−0.333*										−0.312
		(0.190)										(0.227)
Share services			0.020									
			(0.072)									
Labor productivity				0.122								
				(0.114)								
Population density					0.062							
					(0.048)							
Tax revenues						−0.176*						−0.046
						(0.100)						(0.152)
Research & development							−0.638					
							(0.760)					
Gross replacement rate								−0.063*				−0.031
								(0.032)				(0.045)
Tertiary education									0.017			
									(0.044)			
Harmonized unemployment rate										−0.240*		−0.223*
										(0.126)		(0.124)
Share of 25–39 age group in adult population											0.202	
											(0.196)	
Year dummy 1993	0.007	0.021***	0.010	0.011*	0.007	0.011**	0.007	0.013**	0.004	0.010	0.009	0.024**
	(0.007)	(0.007)	(0.007)	(0.006)	(0.007)	(0.005)	(0.007)	(0.005)	(0.010)	(0.006)	(0.008)	(0.010)
Year dummy 2001	0.025**	0.036***	0.025**	0.027***	0.023**	0.023**	0.024**	0.020*	0.020	0.021**	0.030**	0.032**
	(0.011)	(0.012)	(0.010)	(0.010)	(0.011)	(0.009)	(0.010)	(0.010)	(0.013)	(0.009)	(0.014)	(0.012)
Year dummy 2010	0.031**	0.046***	0.031**	0.033***	0.030**	0.025***	0.028**	0.024**	0.022	0.033***	0.042**	0.042**
	(0.011)	(0.014)	(0.011)	(0.011)	(0.012)	(0.009)	(0.011)	(0.009)	(0.019)	(0.011)	(0.019)	(0.017)
Constant	0.094***	0.241***	0.088**	0.081***	0.080***	0.175***	0.104***	0.117***	0.095***	0.125***	0.011	0.289***
	(0.019)	(0.080)	(0.033)	(0.028)	(0.022)	(0.056)	(0.017)	(0.023)	(0.021)	(0.024)	(0.075)	(0.091)
*N*	93	92	92	92	93	92	88	83	85	92	93	83
*R* ^2^	0.22	0.34	0.24	0.28	0.26	0.33	0.19	0.24	0.18	0.29	0.26	0.35

Dependent variable is the (non-agricultural) business ownership rate. Results are from a pooled sample estimation for 1984 (reference year), 1993, 2001 and 2010. Standard errors are reported between brackets and clustered by country

* *p* < 0.10, ** *p* < 0.05, *** *p* < 0.01

Table 8

**Table 8:** The association between religion (Behaving) and the business ownership rate

	Baseline	(1)	(2)	(3)	(4)	(5)	(6)	(7)	(8)	(9)	(10)	Final
Behaving	0.083**	0.047	0.088**	0.078*	0.105**	0.036	0.064*	0.063	0.078*	0.085**	0.065	0.095**
	(0.038)	(0.033)	(0.038)	(0.039)	(0.043)	(0.066)	(0.033)	(0.041)	(0.043)	(0.035)	(0.061)	(0.044)
GDP per capita	−0.026*	−0.023*	−0.038**	−0.069**	−0.024*	−0.026**	−0.019	−0.025**	−0.026	−0.037***	−0.025*	−0.032***
	(0.013)	(0.013)	(0.014)	(0.026)	(0.014)	(0.011)	(0.016)	(0.012)	(0.017)	(0.012)	(0.013)	(0.011)
Female labor share		−0.247										
		(0.175)										
Share services			0.063									
			(0.065)									
Labor productivity				0.137								
				(0.103)								
Population density					0.081*							0.072*
					(0.044)							(0.041)
Tax revenues						−0.146						
						(0.126)						
Research & development							−0.447					
							(0.721)					
Gross replacement rate								−0.058*				−0.043
								(0.030)				(0.029)
Tertiary education									0.021			
									(0.040)			
Harmonized unemployment rate										−0.221*		−0.141
										(0.121)		(0.123)
Share of 25–39 age group in adult population											0.120	
											(0.226)	
Year dummy 1993	0.002	0.010	0.004	0.006	0.002	0.006	0.002	0.008	−0.002	0.005	0.003	0.008
	(0.008)	(0.008)	(0.007)	(0.007)	(0.008)	(0.006)	(0.007)	(0.007)	(0.010)	(0.007)	(0.008)	(0.007)
Year dummy 2001	0.019*	0.027**	0.018	0.020*	0.016	0.020**	0.019*	0.017	0.013	0.017*	0.023	0.014
	(0.011)	(0.012)	(0.011)	(0.011)	(0.011)	(0.010)	(0.011)	(0.011)	(0.014)	(0.010)	(0.015)	(0.010)
Year dummy 2010	0.025**	0.036**	0.023*	0.026**	0.023**	0.024**	0.023*	0.021**	0.015	0.029**	0.032	0.022**
	(0.011)	(0.013)	(0.012)	(0.012)	(0.011)	(0.009)	(0.011)	(0.010)	(0.019)	(0.011)	(0.021)	(0.010)
Constant	0.075***	0.193**	0.047	0.058	0.051*	0.156*	0.087***	0.104***	0.074**	0.104***	0.030	0.091**
	(0.025)	(0.075)	(0.042)	(0.035)	(0.030)	(0.079)	(0.023)	(0.028)	(0.028)	(0.027)	(0.079)	(0.034)
*N*	98	97	97	97	98	97	93	87	90	97	98	87
*R* ^2^	0.26	0.34	0.29	0.32	0.32	0.33	0.22	0.25	0.22	0.32	0.27	0.33

Dependent variable is the (non-agricultural) business ownership rate. Results are from a pooled sample estimation for 1984 (reference year), 1993, 2001 and 2010. Standard errors are reported between brackets and clustered by country

* *p* < 0.10, ** *p* < 0.05, *** *p* < 0.01

Table 9

**Table 9:** Regression coefficients religion dimensions (models controlling for Hofstede’s cultural dimensions (Hofstede et al. 2010))

	Belonging	Believing	Bonding	Behaving
Final model (Table 4)	−0.002 (0.032)	0.059** (0.025)	0.020 (0.043)	0.095** (0.044)
Final model + Power Distance Index (PDI)	0.005 (0.037)	0.056** (0.026)	0.031 (0.046)	0.091* (0.044)
Final model + Individualism versus Collectivism (IDV)	−0.005 (0.035)	0.058** (0.025)	0.025 (0.043)	0.092* (0.046)
Final model + Masculinity versus Femininity (MAS)	−0.004 (0.032)	0.059** (0.026)	0.022 (0.043)	0.110** (0.048)
Final model + Uncertainty Avoidance Index (UAI)	0.005 (0.034)	0.060** (0.026)	0.028 (0.048)	0.086* (0.043)
Final model + Long Term Orientation versus Short Term Normative Orientation (LTO)	−0.008 (0.038)	0.076** (0.037)	0.020 (0.045)	0.081 (0.056)
Final model + Indulgence versus Restraint (IND)	0.005 (0.034)	0.061** (0.025)	0.029 (0.046)	0.094** (0.040)

PDI, IDV, MAS, UAI are missing for Iceland. Dependent variable is the (non-agricultural) business ownership rate. Independent variables are the same as in Table 4. Results are from a pooled sample estimation for 1984 (reference year), 1993, 2001 and 2010. Standard errors are reported between brackets and clustered by country

* *p* < 0.10, ** *p* < 0.05, *** *p* < 0.01

## Footnotes

1 Entrepreneurial behavior refers to behavior associated with the perception, exploitation and creation of new economic opportunities. The occupational notion refers to individuals owning and managing a business on their own account and at their own risk (Wennekers 2006).

2 For instance, belonging to a religious group may provide the necessary social capital to run a large business successfully. However, it may also restrict the size of the business as the customer base is potentially limited to the members of the group. In both cases, there is ample room for entrepreneurship for members of a religious group (either in a large or a smaller business), and a positive relation is expected when measuring the impact of (aggregate level) religion on the individual propensity of entrepreneurship. Hence, the two arguments cannot be distinguished empirically. In contrast, at the country level, these arguments can be distinguished empirically. In particular, if the latter argument (restricted business size) dominates, the relation between religion and the entrepreneurship (business ownership) rate will be stronger as small business entrepreneurship will be more common (i.e., average firm size will be smaller providing more room for small businesses). See also Section 3.2.

3 For instance, Rokeach (1969) finds that religious individuals give higher priority to salvation and less priority to pleasure and independence compared to non-religious individuals.

4 This indirect effect can both be positive and negative. Higher levels of legitimation or moral approval of entrepreneurship may result in favorable institutional context including higher social status for entrepreneurs and favorable tax incentives resulting in higher prevalence rates of entrepreneurship at the aggregate level. The opposite may also hold and a lack of moral approval may be reflected in an institutional context hampering entrepreneurship (Etzioni 1987; Thurik and Dejardin 2012).

5 Our argument relates to the so-called aggregate psychological characteristics approach suggesting that if a society contains more people with values conducive to entrepreneurship, more people will choose the entrepreneurial option (Davidsson 1995; Davidsson and Wiklund 1997; Freytag and Thurik 2007).

6 Inclusion of the agricultural sector makes a substantial difference to measured business ownership rates in many countries. As farm businesses have very different characteristics from non-farm businesses, agricultural workers tend to be excluded from definitions of business ownership or self-employment (Parker 2009, pp. 16–17).

7 Compendia contains harmonized annual data on the number of business owners for 30 OECD countries from 1972 onward. It is one of the few cross-country databases on entrepreneurship rates that exist to date, in addition to the Global Entrepreneurship Monitor and the World Bank Group Entrepreneurship Survey (Marcotte 2013). See http://www.entrepreneurship-sme.eu/ for the data, and see Van Stel (2005) for the harmonization methods applied to make business ownership rates comparable across countries and over time. This database has been used widely in entrepreneurship research (see, among other studies, Armour and Cumming 2008; Carree et al. 2002; Koellinger and Thurik 2012; Noorderhaven et al. 2004; Nyström 2008; Parker et al. 2012; Thurik et al. 2008; Van Praag and Van Stel 2013; Wennekers et al. 2007).

8 Data taken directly from the *OECD Labour Force Statistics* suffer from a lack of comparability across countries and over time. In particular, owner-managers of incorporated businesses (OMIBs) are counted as self-employed in some countries, and as employees in other countries. Also, the raw OECD data suffer from many trend breaks relating to changes in self-employment definitions (Van Stel 2005).

9 Indeed, ANOVA tests show that the mean values (over the 4 waves) for the business ownership rate and the four religion dimensions differ significantly across countries, but the between-wave differences within countries for these variables are not statistically different across countries.

10 This pattern is in line with the main trend in Western countries: A decreasing number of individuals belongs formally to a religious group, but religious beliefs and practices are still present in the population (Lambert 2004, Norris en Inglehart 2004, Maoz and Henderson 2013).

11 Note that in Tables 5, 6, 7 and 8, there is some volatility in the estimates for the religion dimensions across the various auxiliary regressions (i.e., model variants (1)-(10)). This volatility reflects small differences in estimation samples (due to missing observations for the control variable under consideration) as well as the non-inclusion of all relevant control variables as in the final models.

12 The number of observations differs between the four models in Table 4 because of missing values for some of the religion dimensions and for some of the control variables included in the final model specifications. See Tables 5, 6, 7 and 8 in the appendix.

## References

[CR1] Abell P, Crouchley R, Mills C (2001). Social capital and entrepreneurship in Great Britain. Enterp Innov Manag Stud.

[CR2] Aldrich H, Waldinger R (1990). Ethnicity and entrepreneurship. Annu Rev Sociol.

[CR3] Aldrich H, Cater J, Jones T, Mc Evoy D, Velleman P (1985). Ethnic residential concentration and the protected market hypothesis. Soc Forces.

[CR4] Armour J, Cumming D (2008). Bankruptcy law and entrepreneurship. Am Law Econ Rev.

[CR5] Audretsch DB (2007). The entrepreneurial society.

[CR6] Audretsch DB, Bönte W, Tamvada JP (2013). Religion, social class, and entrepreneurial choice. J Bus Ventur.

[CR7] Barnouw V (1985). Culture and personality.

[CR8] Beugelsdijk S, Noorderhaven N (2005). Personality characteristics of self-employed; an empirical study. Small Bus Econ.

[CR9] Blum U, Dudley L (2001). Religion and economic growth: was Weber right?. J Evol Econ.

[CR10] Brüderl J, Preisendörfer P (1998). Network support and the success of newly founded business. Small Bus Econ.

[CR11] Carree MA, Thurik AR, Audretsch DB, Acs ZJ (2010). The impact of entrepreneurship on economic growth. Handbook of entrepreneurship research.

[CR12] Carree MA, Van Stel A, Thurik AR, Wennekers S (2002). Economic development and business ownership: an analysis using data of 23 OECD countries in the period 1976–1996. Small Bus Econ.

[CR13] Carswell P, Rolland D (2007). Religion and entrepreneurship in New Zealand. J Entrepr Commun People Place Glob Econ.

[CR14] Casson M (1982). The entrepreneur: an economic theory.

[CR15] Cohen WM, Klepper S (1992). The tradeoff between firm size and diversity in the pursuit of technological progress. Small Bus Econ.

[CR16] Dana LP (2007). A humility-based enterprising community: the Amish people in Lancaster County. J Enterpr Commun: People Places Glob Econ.

[CR17] Dana LP (2009). Religion as an explanatory variable for entrepreneurship. Int J Entrep Innov.

[CR18] Dana LP, Dana TE (2008). Collective entrepreneurship in a Mennonite community in Paraguay. Lat Am Bus Rev.

[CR19] Davidsson P (1995). Culture, structure and regional levels of entrepreneurship. Entrep Reg Dev.

[CR20] Davidsson P, Honig B (2003). The role of social and human capital among nascent entrepreneurs. J Bus Ventur.

[CR21] Davidsson P, Wiklund J (1997). Values, beliefs and regional variations in new firm formation rates. J Econ Psychol.

[CR22] Dodd SD, Seaman PT (1998). Religion and enterprise: an introductory exploration. Enterp Theory Pract.

[CR23] Etzioni A (1987). Entrepreneurship, adaptation and legitimation: a macro-behavioral perspective. J Econ Behav Org.

[CR24] EVS (2011) European values study 1981–2008, Longitudinal data file. GESIS data archive, Cologne, ZA4804 Data File Version 2.0.0. doi:10.4232/1.11005

[CR25] Freytag A, Thurik AR (2007). Entrepreneurship and its determinants in a cross-country setting. J Evol Econ.

[CR26] Fukuyama F (2001). Social capital, civil society and development. Third World Q.

[CR27] Galbraith CS, Galbraith DM (2007). An empirical note on entrepreneurial activity, intrinsic religiosity and economic growth. J Entrepr Commun People Place Glob Econ.

[CR28] Geertz C, Geertz C (1993). Religion as a cultural system. The interpretation of cultures: selected essays.

[CR29] Glock CY (1962). On the study of religious commitment. Relig Educ.

[CR30] Guiso L, Sapienza P, Zingales L (2006). Does culture affect economic outcomes?. J Econ Perspect.

[CR31] Henley A (2014) Is religion associated with entrepreneurial activity? IZA Discussion Paper, 8111, http://ssrn.com/abstract=2426865

[CR32] Hilary G, Hui KW (2009). Does religion matter in corporate decision making in America?. J Financ Econ.

[CR33] Hill PC, Paloutzian RF, Park CL (2005). Measurement in the psychology of religion and spirituality: current status and evaluation. Handbook of the psychology of religion and spirituality.

[CR34] Hill PC, Pargament KI (2003). Advances in the conceptualization and measurement of religion and spirituality: implications for physical and mental health research. Am Psychol.

[CR35] Hodge D (2003). The intrinsic spirituality scale: a new six-item instrument for assessing the salience of spirituality as a motivational construct. J Soc Serv Res.

[CR36] Hofmann DA (1997). An overview of the logic and rationale of hierarchical linear models. J Manag.

[CR37] Hofstede G, Hofstede GJ, Minkov M (2010). Cultures and organizations: software of the mind.

[CR38] Iannaccone LR (1998). Introduction to the economics of religion. J Econ Lit.

[CR39] Idler EL, Musick MA, Ellison CG, George LK, Krause N, Ory MG, Pargament KI, Powell LH, Underwood LG, Williams DR (2003). Measuring multiple dimensions of religion and spirituality for health research conceptual background and findings from the 1998 General Social Survey. Res Aging.

[CR40] Inglehart R, Baker WE (2000). Modernization, cultural change, and the persistence of traditional values. Am Sociol Rev.

[CR41] Inglehart R, Basanez M, Moreno AM (1998). Human values and beliefs: a cross-cultural sourcebook: political, religious, sexual, and economic norms in 43 societies; findings from the 1990–1993 World Value Survey.

[CR42] Kirzner IM (1973). Competition and entrepreneurship.

[CR43] Koellinger PD, Thurik AR (2012). Entrepreneurship and the business cycle. Rev Econ Stat.

[CR44] König C, Steinmetz H, Frese M, Rauch A, Wang Z (2007). Scenario-based scales measuring cultural orientations of business owners. J Evol Econ.

[CR45] Kumar A, Page JK, Spalt OG (2011). Religious beliefs, gambling attitudes, and financial market outcomes. J Financ Econ.

[CR46] Lambert Y (2004). A turning point in religious evolution in Europe. J Contemp Relig.

[CR47] Licht A (2007). Entrepreneurial spirit and what the law can do about it. Comp Labor Law Policy J.

[CR48] Lucas RE (1978). On the size distribution of business firms. Bell J Econ.

[CR49] Maoz Z, Henderson EA (2013). The world religion dataset, 1945–2010: logic, estimates, and trends. Int Interact.

[CR50] Marcotte C (2013). Measuring entrepreneurship at the country level: a review and research agenda. Entrep Reg Dev.

[CR51] Nelson R (2006). Evolutionary social science and universal Darwinism. J Evol Econ.

[CR52] Noorderhaven NG, Thurik AR, Wennekers S, Van Stel AJ (2004). The role of dissatisfaction and per capita income in explaining self-employment across 15 European countries. Enterp Theory Pract.

[CR53] Norris P, Inglehart R (2004). Sacred and secular: religion and politics worldwide.

[CR54] North DC (1991). Institutions. J Econ Perspect.

[CR55] Noseleit F, Freytag A, Thurik AR (2010). The entrepreneurial culture: guiding principles of the self-employed. Entrepreneurship and culture.

[CR56] Noussair CN, Trautmann ST, Van de Kuilen G, Vellekoop N (2013). Risk aversion and religion. J Risk Uncertain.

[CR57] Nyström K (2008). The institutions of economic freedom and entrepreneurship: evidence from panel data. Public Choice.

[CR58] Parboteeah KP, Walter SG, Block JH (2015). When does Christian religion matter for entrepreneurial activity? The contingent effect of a country’s investment into knowledge. J Bus Ethics.

[CR59] Parker SC (2009). The economics of entrepreneurship.

[CR60] Parker SC, Congregado E, Golpe A (2012). Testing for hysteresis in entrepreneurship in 23 OECD countries. Appl Econ Lett.

[CR61] Portes A, Lesser EL (2000). Social capital: its origins and applications in modern sociology. Knowledge and social capital.

[CR62] Putnam RD (2000). Bowling alone: the collapse and revival of American community.

[CR63] Rietveld CA, Van Burg E (2014). Religious beliefs and entrepreneurship among Dutch protestants. Int J Entrepr Small Bus.

[CR64] Roccas S (2005). Religion and value systems. J Soc Issues.

[CR65] Rokeach M (1969). Value systems in religion. Rev Relig Res.

[CR66] Saroglou V (2002). Beyond dogmatism: the need for closure as related to religion. Ment Health Relig Cult.

[CR67] Saroglou V (2011). Believing, bonding, behaving, and belonging. The big four religious dimensions and cultural variation. J Cross-Cult Psychol.

[CR68] Saroglou V, Delpierre V, Dernelle R (2004). Values and religiosity: a meta-analysis of studies using Schwartz’s model. Personal Individ Differ.

[CR69] Schumpeter JA (1934). The theory of economic development.

[CR70] Schwartz SH (1992) Universals in the content and structure of values: theoretical advances and empirical tests in 20 countries. In: Zanna MP (ed) Advances in experimental social psychology. Academic, Orlando 25: pp.1–65

[CR71] Schwartz SH, Huisman S (1995). Value priorities and religiosity in four Western religions. Soc Psychol Q.

[CR72] Sørensen JB, Stuart TE (2000). Aging, obsolescence, and organizational innovation. Adm Sci Q.

[CR73] Tarakeshwar N, Stanton J, Pargament KI (2003). Religion an overlooked dimension in cross-cultural psychology. J Cross-Cult Psychol.

[CR74] Thurik AR, Dejardin M, Van Gelderen M, Masurel E (2012). Culture and entrepreneurship. Entrepreneurship in context.

[CR75] Thurik AR, Carree MA, Van Stel AJ, Audretsch DB (2008). Does self-employment reduce unemployment?. J Bus Ventur.

[CR76] Tomes N (1985). Religion and the earnings function. Am Econ Rev.

[CR77] Van Praag CM, Van Stel A (2013). The more business owners, the merrier? The role of tertiary education. Small Bus Econ.

[CR78] Van Praag CM, Versloot PH (2007). What is the value of entrepreneurship? A review of recent research. Small Bus Econ.

[CR79] Van Stel A (2005). COMPENDIA: harmonizing business ownership data across countries and over time. Int Entrepr Manag J.

[CR80] Verbit MF (1970). The components and dimensions of religious behavior: toward a reconceptualization of religiosity.

[CR81] Weber M (1930). The Protestant ethic and the spirit of capitalism.

[CR82] Wennekers ARM (2006). Entrepreneurship at country level: economic and non-economic determinants.

[CR83] Wennekers S, Thurik AR, Van Stel A, Noorderhaven N (2007). Uncertainty avoidance and the rate of business ownership across 21 OECD countries, 1976–2004. J Evol Econ.

[CR84] Wennekers S, Van Stel A, Carree M, Thurik AR (2010). The relationship between entrepreneurship and economic development: is it U-shaped?. Found Trends Entrepr.

[CR85] Williamson OE (2000). The new institutional economics: taking stock, looking ahead. J Econ Lit.

[CR86] Zelekha Y, Avnimelech G, Sharabi E (2014). Religious institutions and entrepreneurship. Small Bus Econ.

